# High Serpin Family A Member 10 Expression Confers Platinum Sensitivity and Is Associated With Survival Benefit in High-Grade Serous Ovarian Cancer: Based on Quantitative Proteomic Analysis

**DOI:** 10.3389/fonc.2021.761960

**Published:** 2021-11-23

**Authors:** Wenwen Guo, Xue He, Jing Ni, Liya Ma, Xianzhong Cheng, Congyang Wang, Xiaoxiang Chen, Yan Wang

**Affiliations:** ^1^ Department of Pathology, The Second Affiliated Hospital of Nanjing Medical University, Nanjing, China; ^2^ Department of Gynecologic Oncology, Jiangsu Cancer Hospital, Jiangsu Institute of Cancer Research, The Affiliated Cancer Hospital of Nanjing Medical University, Nanjing, China

**Keywords:** high-grade serous ovarian cancer, platinum sensitivity, platinum resistance, extracellular matrix, SERPINA10

## Abstract

This study aims to identify differentially expressed proteins related with platinum sensitivity and to find biomarkers for predicting platinum response and survival outcomes in patients with high-grade serous ovarian cancer (HGSOC). Eligible HGSOC patients were divided into platinum-sensitive and platinum-resistant groups according to platinum-free interval (PFI). Tissue protein lysates from tumor tissues were subjected to an in-solution tryptic digest followed by tandem mass tag (TMT) labeling of the resulting peptides and mass spectrometric analysis. Candidate proteins were identified using differentially expressed protein and gene set enrichment analysis (GSEA) and confirmed by immunohistochemistry (IHC), and their survival relevance was evaluated in The Cancer Genome Atlas (TCGA) ovarian cancer cohort. The results showed that there was a significant difference in the protein expression profiling between the two patient groups. In the GSEA model, a gene set of 239 extracellular matrix (ECM)-related proteins was significantly enriched in the platinum-sensitive group [normalized enrichment score (NES) = 3.82, q < 10^−5^], and this finding was confirmed in TCGA ovarian cancer cohort. Interestingly, an ECM-related gene expression, *serpin family A member 10* (*SERPINA10*), was identified to be significantly positively correlated with overall survival (OS) and progression-free survival (PFS) in TCGA ovarian cancer cohort (all *p* < 0.05). IHC results demonstrated that HGSOC patients with high SERPINA10 expression had longer PFI than the patients with low SERPINA10 expression (9 vs. 5 months, *p* = 0.038), and the SERPINA10 expression had an area under the receiver operating characteristic curve (AUC) value of 0.758 (95% CI = 0.612–0.905; *p* = 0.005) to discriminate the platinum-sensitive group from the platinum-resistant group. In conclusion, the results suggested that SERPINA10 could be a promising biomarker for predicting the response and survival in platinum-based chemotherapy of HGSOC.

## Introduction

Ovarian cancer is the most lethal gynecologic malignancy (1), of which high-grade serous ovarian cancer (HGSOC) is the most prevalent and aggressive histologic subtype and accounts for 70%–80% of deaths (2, 3). Despite recently reported significant progress in poly(adenosine diphosphate [ADP]-ribose) polymerase (PARP) inhibitor for ovarian cancer treatment (4), the standard therapy for HGSOC still remains cytoreductive surgery and platinum-based chemotherapy (1). The major obstacle to clinical application of platinum-based chemotherapy is the resistance by tumor cells, which can result in the failure of therapy, relapse, and even death. In clinical practice, response to platinum-containing anticancer drugs is an essential prognostic determinant for survival in HGSOC patients. Therefore, it is necessary to explore relevant biomarkers in predicting the response and targets in overcoming resistance to the platinum-containing drugs in the treatment of ovarian cancer.

HGSOC patients who progress during initial treatment of platinum-based chemotherapy or completely alleviated after initial treatment that includes cytoreductive surgery and the platinum-based chemotherapy but recur within 6 months are defined as platinum-resistant recurrent (PRR) ovarian cancer patients (5, 6), and platinum-sensitive recurrent (PSR) patients initially respond to first-line platinum-based chemotherapy after cytoreductive surgery and do not relapse within 6 months from the last platinum-based treatment (5, 6). Although multiple mechanisms have been proven to be involved in the regulation of platinum sensitivity in ovarian cancer such as uptake, transport and metabolism of the platinum-containing drugs, DNA repair of platinum-induced damages, cancer stem cells (CSCs), and epithelial-to-mesenchymal transition (EMT) (7–12), the underlying influencing factors of platinum sensitivity are not fully clarified yet and need more in-depth study. In the present study, in order to explore the molecular basis of platinum resistance and further determine a potential biomarker for platinum sensitivity prediction, we analyzed the differentially expressed proteins of tumor tissues from platinum-sensitive and platinum-resistant HGSOC patient groups by proteomic analysis and investigated their possible clinical relevance and performance for prediction of platinum sensitivity in ovarian cancer treatment. The results suggested that a set of extracellular matrix (ECM)-related proteins might be the molecular basis of a platinum-sensitive phenotype of HGSOC and identified a promising protein marker involved in the ECM that could predict platinum sensitivity of HGSOC patients.

## Materials and Methods

### Patient Samples

The study procedure was approved by the local ethics committee of the Nanjing Medical University (reference number: NJMU 2021-432) and was performed in accordance with the principles of the Declaration of Helsinki. Tumor samples, including snap-frozen and formalin-fixed paraffin-embedded (FFPE) tissues, were obtained from primary focus of HGSOC. Enrolled patients received platinum-based regimens (**Table S1**) after primary debulking surgery with informed consent. Fresh tissue samples for whole proteomic analysis were collected from cohort 1 patients at the time of surgery. Excised tumor tissues were confirmed by senior oncologists (JN and XXC) and pathologists (WG and YW) and immediately dissected, snap-frozen, and stored in liquid nitrogen. The FFPE tissues from 41 unrelated HGSOC patients of cohort 2 were used to verify the protein expressions of candidate genes in the proteomic results. Based on clinical information, eligible HGSOC patients were allocated into PSR and PRR groups, according to the platinum-free interval (PFI) calculated from the last platinum-based chemotherapy to the time of recurrence (6).

### Liquid Chromatography–Tandem Mass Spectrometry

Protein quantification was applied by liquid chromatography–tandem mass spectrometry (LC-MS/MS)-based proteomic analysis (PTM Biolabs). Protein samples were extracted from snap-frozen tumor tissues. Briefly, the tumor tissue was grinded by liquid nitrogen into cell powder and then transferred to a 5-ml centrifuge tube. After that, four volumes of lysis buffer (8 M of urea, 1% protease inhibitor cocktail) was added, followed by sonication three times on ice using a high-intensity ultrasonic processor (Scientz). The supernatant was collected by centrifugation at 12,000 *g* for 10 min at 4°C, and the protein concentration was determined with bicinchoninic acid (BCA) assay (Sigma). Then, extracted proteins were digested into peptides through trypsin. Tryptic peptides were processed using tandem mass tag (TMT) labeling kit according to the manufacturer’s protocol and then fractionated to reduced sample complexity by high pH reversed-phase high-performance LC (HPLC). Fractions were then analyzed and performed using an EASY-nLC 1000 ultra-HPLC (UHPLC) system coupled to a Q Exactive Plus Hybrid Quadrupole-Orbitrap mass spectrometer (Thermo). Finally, fragment ion spectra acquired from LC-MS/MS analysis were assigned peptide sequences based on database comparison, and protein levels were quantified.

### Exploratory Data Analysis

The (dis)similarities of proteomic data between the two groups of patients were examined by Pearson’s correlation analysis and principal component analysis (PCA) through Corrplot and ClustVis R packages, respectively. Hierarchical clustering for Z-score normalized abundance values of all identified protein in proteomic analysis was performed using pheatmap R package with Euclidean distance and complete linkage algorithm. To identify proteomic changes, differentially expressed proteins between the two groups were screened with *p*-value less than 0.05 (Student’s t-tests), and the fold change of more than or equal to 2 was considered as the threshold.

To explore the biological processes responsible for the platinum sensitivity, we used gene set analysis (GSA). The GSA was performed using set enrichment analysis (GSEA) v4.1.0 application on the proteomic data. Phenotypes were defined by PFI and divided into the PSR and PRR groups. Hallmarks of gene set (h.all.v7.4.symbols.gmt) were used as input to GSEA to determine gene enrichment or depletion for platinum sensitivity [false discovery rate (FDR) <0.001]. This was followed by identifying candidate proteins implicated in platinum sensitivity of HGSOC shared between the upregulated part of the differentially expressed proteins and the ranked gene set with the highest normalized enrichment score (NES) of GSEA. A Venn diagram was generated to show those shared candidate proteins by Venn Diagram package of R software.

We obtained those candidate protein-coding gene expression data of RNA sequencing (RNAseq) and the corresponding clinical information from The Cancer Genome Atlas (TCGA) dataset (https://portal.gdc.com). Subsequently, clinical relevance of those candidate protein-coding gene expressions in TCGA ovarian cancer cohort was determined by univariate Cox proportional hazard analyses to estimate the hazard ratio (HR) and 95% CI of the overall survival (OS) and progression-free survival (PFS). The Kaplan–Meier survival curves with log-rank tests were made for further comparison of the survival difference between the groups of high and low candidate protein-coding gene expression, and estimation of time-dependent area under the receiver operating characteristic (ROC) curves (AUCs) for survival data and candidate protein-coding gene signatures was performed using time-ROC R package in TCGA ovarian cancer cohort.

### Immunohistochemistry Staining

Immunohistochemistry (IHC) analysis was conducted on samples collected from 41 HGSOC patients. It was done according to standard protocols using antibodies against IGFBP4 (ProteinTech), TGFBI (ProteinTech), and serpin family A member 10 (SERPINA10) (ProteinTech); and cytoplasmic staining was considered as positive. Staining was assessed by three independent pathologists (WG, XH, and YW). The immunoreactive score (IRS) gave a range of 0–12 as a product of multiplication between positive cell proportion score (0, no positive cells; 1, <10% of positive cells; 2, 10–50% positive cells; 3, 51%–80% positive cells; and 4, >80% positive cells) and staining intensity score (0, no color; 1, mild staining; 2, moderate staining; 3, intense staining) (13).

### Statistical Analysis

Data were presented as the mean ± SD or median (range). Comparisons of continuous variables between groups were performed using GraphPad Prism application (v8.0.2.263) with the Mann–Whitney U test or Student’s t-test as indicated. ROC curve for platinum-sensitive prediction and related AUC were calculated and constructed in the GraphPad Prism application. In all cases, the differences were considered statistically significant when a two-side *p*-value was lower than 0.05.

## Results

### Proteomic Analysis Reveals a Significant Comparative Difference in Protein Expression Profiling Between Platinum-Sensitive and Platinum-Resistant Tumors

To explore gene signature-related platinum sensitivity of HGSOC tumors, the proteomic analysis was performed in cohort 1 subjects. The cohort 1 with age 44 to 68 years was unrelated HGSOC patients, and all patients had advanced International Federation of Gynecology and Obstetrics (FIGO) stage (IIIC or IV stage) (**Table S1**). It consisted of two patient groups (platinum-sensitive and platinum-resistant) classified by PFI (above or below 6 months).

**Figure 1:**
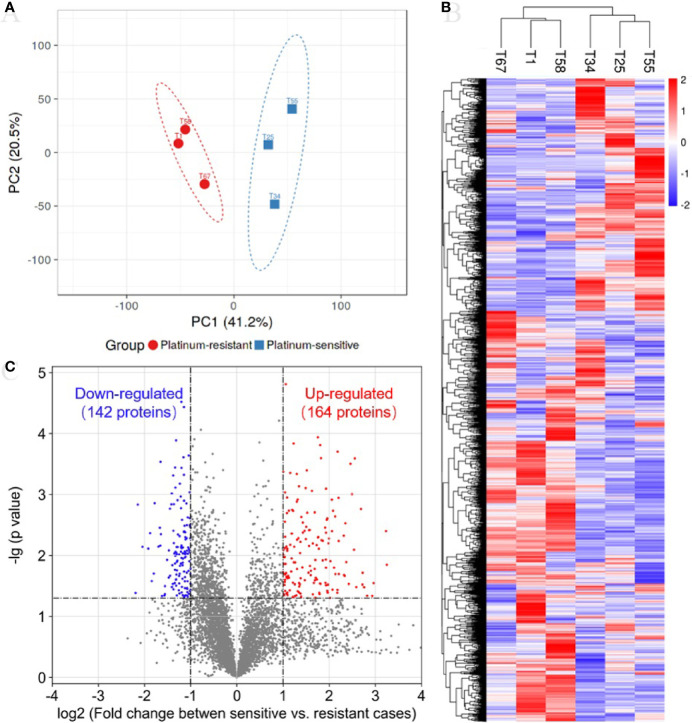
Proteomic analysis reveals a difference protein expression profiling between platinum-sensitive and platinum-resistant tumors. **(A)** Visualization of the first two principal components (PCs) from principal component analysis (PCA) separating samples based on the Z-score normalized quantification for identified proteins. The tissues from platinum-sensitive (blue square) and platinum-resistant (red circle) patients are labelled with different shapes and colors, and a 95% confidence ellipse for each group of samples was drawn. **(B)** Hierarchical clustering and heatmap of Z-score normalized expression values of all identified proteins in platinum-sensitive and platinum-resistant tumors. Red, high abundance; and blue, low abundance. **(C)** Volcano plot for the comparison between the platinum-sensitive and platinum-resistant tumors. The cutoff values of fold change ≥2 and *p*-value < 0.05 were utilized to identify differentially expressed proteins. Non-changed proteins are shown as gray dots, upregulated proteins as red dots, and downregulated proteins as blue dots.

In the TMT quantification proteomics, a total of 44,910 peptides and 6,351 proteins were identified with 1% FDR, and quantifiable proteins were 5,326 (**Table S2**). To determine whether samples clustered based on the platinum sensitivity, we performed a PCA on the quantitative proteins of proteomics across all samples. The first and secondary principal components (PC1 and PC2) separated all samples, contributing 41.2% and 20.5% explanation of variance, respectively (**Figure 1A**). The PC1 clearly separated platinum-sensitive patients from the platinum-resistant group (**Figure 1A**), highlighting the differences of expressed proteins associated with response to platinum chemotherapy in ovarian cancer. The abundance of proteins estimated by their mass spectrometry signal intensities has an obviously positive correlation in samples with the same response to platinum chemotherapy (**Figure S1**). This result supported the (dis)similarities between the proteomic measurements of the samples from the two groups. Additionally, hierarchical clustering analysis showed that there was clearly different protein expression profiling between the two groups (**Figure 1B**). Compared with those from the platinum-resistant group, there were a total of 306 differentially expressed proteins (164 upregulated and 142 downregulated proteins) in the tumors from the platinum-sensitive group (**Figure 1C**). The heatmap of the differentially expressed proteins is shown in **Figure S2**, which indicated that those expressed proteins could correctly distinguish the two kinds of tumors.

### Enriched Expression of Extracellular Matrix-Related Proteins Associated With Platinum-Sensitive Phenotype of High-Grade Serous Ovarian Cancer

**Figure 2:**
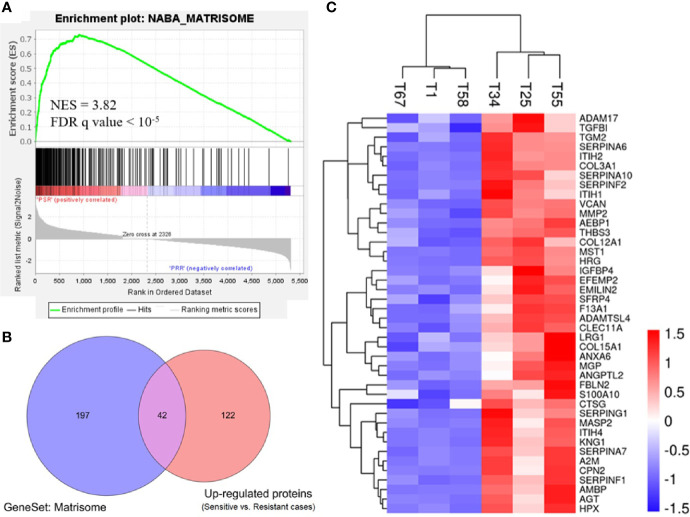
Enriched expression of extracellular matrix-related proteins in the platinum-sensitive tumors. **(A)** Gene set enrichment analysis (GSEA) of a gene set for extracellular matrix-related proteins in the proteomic data. NES, normalized enrichment score; FDR, false discovery rate; PSR, platinum-sensitive recurrent high-grade serous ovarian cancer (HGSOC). PRR, platinum-resistant recurrent HGSOC. **(B)** Overlapping proteins of interest between the extracellular matrix gene cluster (matrisome) of 239 proteins and the upregulated proteins (platinum-sensitive tumors *vs.* platinum-resistant tumors). **(C)** Heatmap of Z-score normalized expression abundance values of the 42 overlapping proteins of interest hierarchically clustered with Euclidean distance matrix and complete linkage. Red, high abundance; and blue, low abundance.

To gain biological insights into the platinum sensitivity of ovarian cancer, we first identified 276 gene set hallmarks associated with platinum sensitivity (FDR q value <0.001, **Table S3**) by GSEA using the signal-to-noise measure based on the proteomic data (platinum-sensitive vs. platinum-resistant). Of these hallmarks, a gene set of 239 ECM-related proteins and the matrisome cluster of 110 proteins (matrisome was referred to as a set of ECM-associated proteins) were the most associated hallmark of GSEA, and they were significantly enriched in the platinum-sensitive group with NES 3.82 (q < 10^−5^) (**Figure 2A** and **Table S3**). To confirm this result, we further examined the gene set of 239 ECM-related genes in publicly available TCGA gene expression data of ovarian cancer, and we observed highly consistent correlation that the expressions of those genes were positively related to a platinum-sensitive phenotype of ovarian cancer (NES = 2.466, FDR q value <0.0001; **Figure S3**).

### Prognostic and Predictive Values of Candidate Protein Expressions for Platinum Sensitivity and Survival of High-Grade Serous Ovarian Cancer Patients

**Figure 3:**
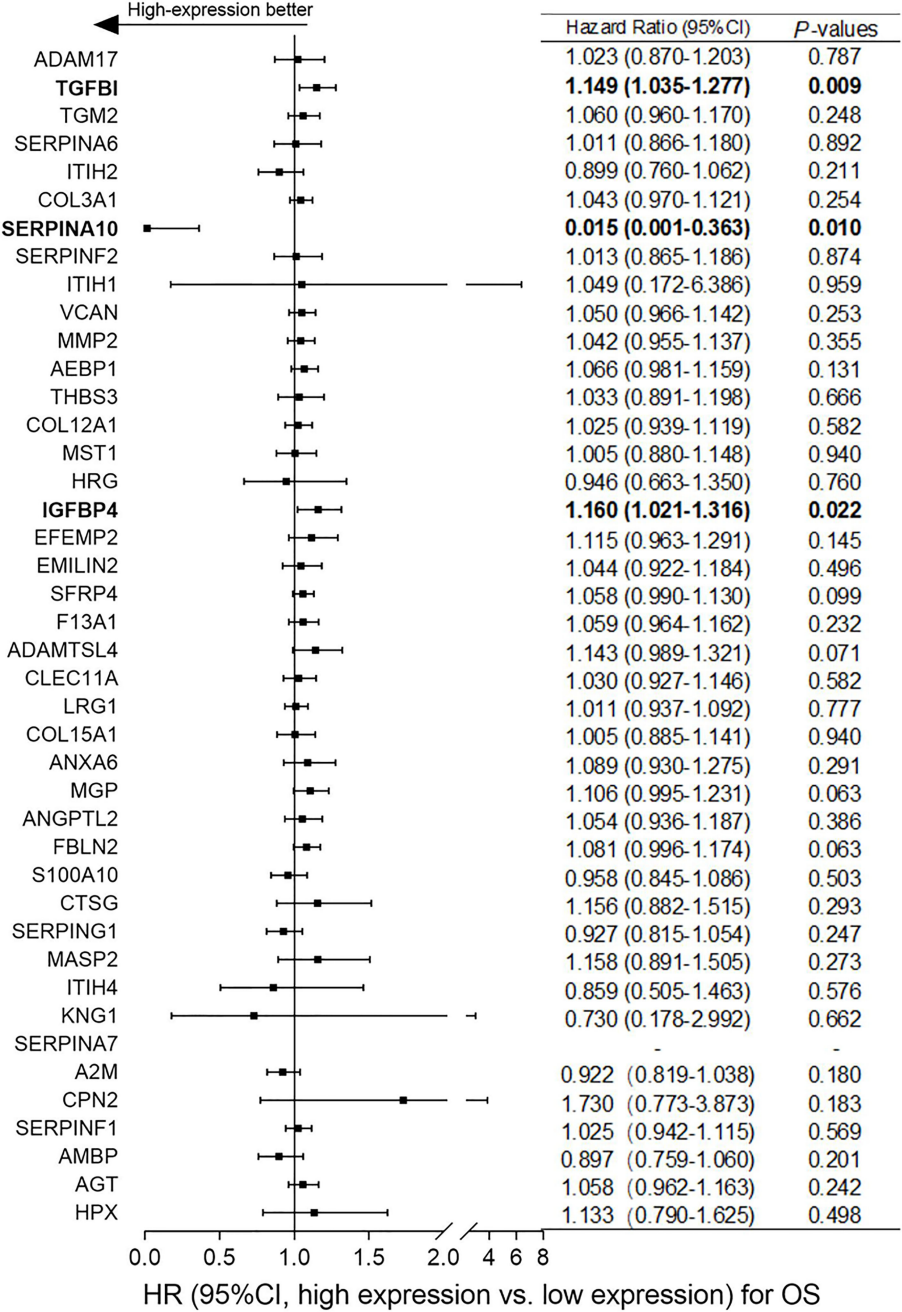
Correlation analyses of mRNA expressions of the 42 overlapping candidate proteins and overall survival (OS) of ovarian cancer patients in The Cancer Genome Atlas (TCGA) dataset. According to the median expression level of each candidate protein, patients were assigned to high-expression and low-expression groups. Normalized expression values of RNA-sequencing data and survival data of ovarian cancer were obtained from TCGA dataset. Hazard ratio (HR) with 95% CI and *p*-values were generated by univariate Cox proportional hazard regression. *p* < 0.05 was considered statistically significant. “High-expression better” means that ovarian cancer patients with high expression of candidate genes had better overall survival (OS).

Based on the differentially expressed proteins between platinum-sensitive and platinum-resistant tumors, 42 proteins in the platinum-sensitive tumors were chosen as candidate genes (**Figures 2B, C**) because they had been ranked in the list of the above platinum-sensitive phenotype-associated ECM protein cluster through GSEA. The associations of those candidate gene expressions with survival were estimated in TCGA ovarian cancer cohort, and the Cox proportional hazard analysis showed that most of the candidate gens were not related to survival benefits in TCGA ovarian cancer cohort. Survival analysis uncovered that *SERPINA10* expression could be a prognostic factor for OS and PFS and showed that high *SERPINA10* expression was correlated with longer OS and lower risk of disease progression (**Figures 3** and **Figure S4**). Contrary to the hypothesis that those upregulated candidate genes might be a survival benefit for ovarian cancer, high *IGFBP4* and *TGFBI* gene expressions were associated with decreased OS rather than prolonged survival (**Figure 3**).

**Figure 4:**
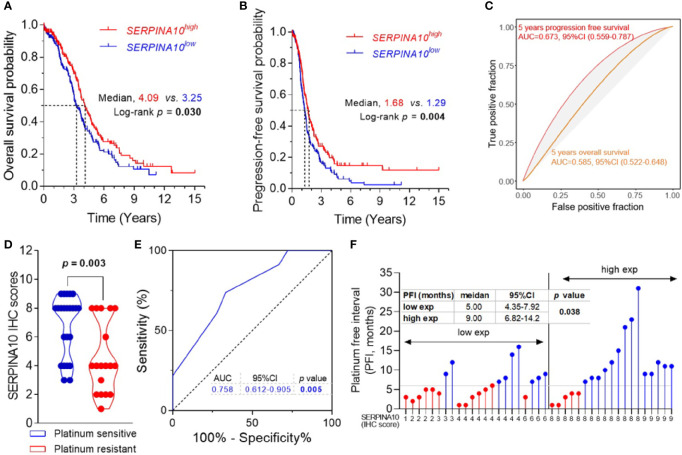
Prognostic and predictive value of SERPINA10 expression for platinum sensitivity and survival of high-grade serous ovarian cancer (HGSOC) patients. **(A, B)** Kaplan–Meier analysis of overall survival (OS) and progression-free survival (PFS) for The Cancer Genome Atlas (TCGA) ovarian cancer cohort, stratified by median value of *SERPINA10* mRNA expressions. Medians of survival (OS and PFS) and log-rank *p*-Values were indicated, respectively. **(C)** Time-dependent receiver operating characteristic (ROC) curves of *SERPINA10* expression for the predictions of 5-year OS and PFS. Expression data of RNA-sequencing and survival data of ovarian cancer were acquired from TCGA dataset. **(D)** Immunohistochemical staining of SERPINA10 expression in HGSOC patients. *p*-Values were calculated by Mann–Whitney test. **(E)** ROC curves of SERPINA10 expression in predicting platinum-sensitivity status of HGSOC patients who were receiving platinum-based chemotherapy treatment after cytoreductive surgery. The AUC was the highest for the SERPINA10 expression (AUC = 0.758; 95% CI = 0.612–0.905; *p* = 0.005). **(F)** Correlation analysis of SERPINA10 expression and platinum-free interval (PFI) in HGSOC patients. Grouping of HGSOC patients according to their immunoreactive scores (cutoff = 6) into low exp (n = 22) or high exp (n = 19). *p*-Value was calculated by Mann–Whitney test. AUC, area under the curve.

Kaplan–Meier curves of OS and PFS stratified by medians of *SERPINA10* mRNA expressions in TCGA dataset revealed that patients with high *SERPINA10* expression had significantly prolonged OS and PFS than whose tumors with low *SERPINA10* expression (**Figures 4A, B** and **Table S4**). Meanwhile, time–ROC curves of the three genes for 5-year OS and PFS predictions were plotted in TCGA ovarian cancer cohort, and AUC of the 5-year OS outcome for *SERPINA10* expression was 0.585 (95% CI = 0.522–0.648) (**Figure 4C**). For predicting 5-year PFS outcome of *SERPINA10* expression, its AUC value was 0.673 (95% CI = 0.559–0.787) (**Figure 4C**).

To validate three candidates, IGFBP4, TGFBI, and SERPINA10, their expressions, and their prognostic values in HGSOC patients, we assessed the three protein expressions by IHC analysis in 41 FFPE tissues of HGSOC patients. The age of 41 HGSOC patients ranged from 42 to 77 years with the median age of 56.0 years, and 19.5% (8/41) patients had FIGO stage III disease, and 80.5% (33/41) had FIGO stage IIIC or IV disease (**Table S1**). IHC staining showed that expressions of IGFBP4, TGFBI, and SERPINA10 proteins were detected in para-tumoral normal cells and tumor cells that were mostly located in the extracellular and cytosol compartments (**Figure S5**). The quantitative results of IHC staining indicated that there were no statistical differences of IGFBP4 and TGFBI IHC scores between platinum-sensitive and platinum-resistant groups (**Figure S6A**). It was important that SERPINA10 IHC score of the platinum-sensitive group was notably higher than that of platinum-resistant group (**Figure 4D**). Results of ROC analysis are shown in **Figure 4E**, given an AUC value of 0.758 with 95% CI of 0.612 to 0.905 (*p* = 0.005) for the predictive effect of the SERPINA10 signature on platinum sensitivity, which was higher than 0.7 suggested for a successful prediction in platinum sensitivity of HGSOC patients. In addition, we observed that HGSOC patients with high SERPINA10 expression (IHC score greater than 6) had longer PFI than patients with low SERPINA10 expression (IHC score less than 8) (9 vs. 5 months, *p* = 0.038) (**Figure 4F**).

## Discussion

Ovarian cancer accounts for roughly 4% of all cancer diagnoses among women worldwide (14), but it is the chief death cause in gynecological cancer (2). Although PARP inhibitors had changed the treatment mode of ovarian cancer, the standard treatment of HGSOC is still cytoreductive surgery and platinum-based chemotherapy (4, 15). Clinically, approximately 80% of advanced HGSOC patients continue to have poor long-term survival mainly due to platinum resistance and tumor recurrence (5, 15). Numerous studies have been devoted to determine the molecular biological mechanism of platinum resistance and recurrence. But so far, research on overcoming platinum-resistance strategies and biomarker for predicting response to platinum-based chemotherapy is still needed in the clinical management of ovarian cancer. In present study, we found that there was a significant difference of protein expression profiling between platinum-sensitive and platinum-resistant tumors by proteomic analysis, which would be a molecular basis in biomarker screening for predicting response to platinum-based chemotherapy and in resolving of mechanism of platinum resistance. A total of 306 differentially expressed proteins (164 upregulated and 142 downregulated proteins) were observed between platinum-sensitive and platinum-resistant tumors in proteomic analysis. More interestingly, results of GSEA based on the proteomic data showed that a set of ECM-related proteins (including matrisome proteins) was the most interesting gene cluster significantly enriched in the platinum-sensitive HGSOC group. A similar result was seen in GSEA of gene expression data of TCGA ovarian cancer cohort, and we identified that the above-observed gene set of 239 ECM-related genes was positively correlated with a platinum-sensitive phenotype (NES = 3.82, FDR q value <10^−5^). ECM is a three-dimensional structure composed of glycoproteins, collagens, and proteoglycans and crucial for maintaining cellular homeostasis (16, 17), and the ECM has been implicated in the regulation of tumor cell behavioral and response to tumor therapeutics (hormonal and chemotherapeutics) (11, 18–20). Especially, the tumor-associated matrisome signatures, known as matrix index (MI), could be a predictor for poor prognosis in different kinds of solid tumors (19). Additionally, Barkan et al. reported that ECM could modulate tumor dormancy and serve as a “gatekeeper” in transformation of tumor cells from quiescence to proliferation and has potential to positively contribute on tumor recurrence and chemotherapy resistance (21). Etemadmoghadam et al. suggested that increased expression of ECM-related genes and ECM deposition probably were notable molecular features of primary resistance to chemotherapy in ovarian cancer (22). Clearly, those results from studies on GSA are contrary to the findings in the GSA model of the present study, and they found that ECM gene clusters were related to resistance to chemotherapy in the ovarian cancer (20, 22). However, further ingenuity pathway analysis (IPA) suggested that those published ECM clusters involved transforming growth factor beta 1 (TGF-beta) signaling pathway, and that might be a key contributor to EMT and resistance to chemotherapy with paclitaxel- and platinum-containing drugs (20). Similarly, in the GSEA on the proteomic data, we observed that the molecular signatures of TGF-beta-induced signaling (REACTOME_SIGNALING_BY_TGF_BETA_RECEPTOR_COMPLEX) and genes upregulated by TGF-beta (KARLSSON_TGFB1_TARGETS_UP) were significantly enriched in platinum-resistant tumors with NES −1.67 (q = 0.048) and −1.98 (q = 0.003), respectively (**Table S5**). These results were consistent with the activation of TGF-beta signaling participated in and might therefore serve as a biomarker for resistance to chemotherapy.

Several studies described above have suggested that a TGF-beta signaling activation-related ECM of tumor cells can play a role in cell adhesion-mediated drug resistance (CAM-DR), such as resistance to platinum drugs (20). However, Ahmed et al. elucidated that an ECM-related gene, TGF-beta-induced (TGFBI), expression was significantly increased in drug-sensitive ovarian cancer patients compared with drug-resistant patients, and *in vitro* experiments showed that TGFBI-associated integrin signaling pathway could be related to paclitaxel sensitivity of ovarian cancer and breast cancer cell lines (23). Wang et al. also demonstrated that TGFBI was frequently methylated in paclitaxel-resistant ovarian cancer cells (24), and this hypermethylation was in line with the above-observed molecular phenotype of low TGFBI expression (24). In the present study, we found that TGFBI was a member of an overlap of 42 candidate proteins between the upregulated proteins and the gene set of 239 ECM-related proteins. It is speculated that high expressed TGFBI may be related to the survival benefit for ovarian cancer patients on chemotherapy treatment. However, Cox proportional hazard analysis showed the high TGFBI expression was negatively correlated with OS in TCGA ovarian cancer cohort, and its expression had no significant predictive power of platinum sensitivity in IHC verification (**Figure S6B**). These results were inconsistent, probably because of the small sample size, different histological types and stages, therapeutic regimen, and response evaluation criteria across those studies as well as the complexity and diversity of platinum-resistance mechanisms for ovarian cancer. In particular, the role of ECM in platinum resistance of solid tumor cells needs further examination. As mentioned, the gene set of large ECM-related genes was positively correlated with a platinum-sensitive phenotype of HGSOC, which implied that their expressions may be related to maintenance of an epidermoid state of ovarian cancer cells that could render sensitivity to platinum chemotherapy (25).

In the 42 candidate proteins, we identified SERPINA10, an ECM-related protein, which was significantly positively correlated with OS and PFS in TCGA ovarian cancer cohort. SERPINA10, known as protein Z-dependent protease inhibitor, is a member of the serpin superfamily of proteinase inhibitors, and most of them are located in the extracellular space (26). It has been previously suggested that overexpression of SERPINA10 in tumor tissues of pancreatic endocrine tumors (PETs) and small bowel neuroendocrine tumors (SBNETs) might play an important role in tumor progression and metastasis (27–29) and could be a candidate marker for disease diagnosis and treatment. In this study, the result showed that SERPINA10 expressed higher in the platinum-sensitive HGSOC tissues than in platinum-resistant HGSOC tissues. Furthermore, immunohistological examination and ROC analysis determined that SERPINA10 staining intensity was positively correlation with PFI of HGSOC patients and could be used as an index for predicting platinum sensitivity of HGSOC patients. It is necessary to further explore its role for response to platinum-containing drug in ovarian cancer.

In summary, the results suggested that a gene set of large ECM-related proteins is the most interesting gene cluster enriched in the platinum-sensitive tumors. Further study is warranted to investigate their roles for chemoresistance of ovarian cancer. More importantly, we have observed that the protein level of SERPINA10, an ECM-related protein, is significantly positively correlated with survival (OS and PFS) and PFI in HGSOC patients, and the results demonstrated that SERPINA10 may be a potentially promising protein marker for predicting and monitoring the response to chemotherapy in HGSOCs.

## Data Availability Statement

The original contributions presented in the study are publicly available. The dataset PXD028225 can be found here, http://proteomecentral.proteomexchange.org/cgi/GetDataset?ID=PXD028225.

## Ethics Statement

The study procedure was approved by the local ethics committee of the Nanjing Medical University and was performed in accordance with the principles of the Declaration of Helsinki. The committee’s reference number was Ethical Committee of Nanjing Medical University 2021-432. The patients/participants provided their written informed consent to participate in this study.

## Author Contributions

WG, XH, and JN designed the study, recruited/evaluated the patients, performed most of the laboratory experiments, analyzed the data, and drafted the manuscript. LM and CW performed some of the laboratory procedures (IHC) and analyzed the data. XZC collected the cases and analyzed the data. XXC and YW designed the study and are responsible for the entire manuscript. All authors contributed to the article and approved the submitted version.

## Funding

This work was supported by the Six Major Talent Summit of Jiangsu (2018-WSW-063), Youth Medical Talent of Jiangsu Province (QNRC2016665), 789 Outstanding Talent Program of the Second Affiliated Hospital of Nanjing Medical University (789ZYRC202080122), Natural Science Foundation of Youth Fund Projects of Jiangsu Province (SBK2021040731), and National Natural Science Foundation of China (81472441).

## Conflict of Interest

The authors declare that the research was conducted in the absence of any commercial or financial relationships that could be construed as a potential conflict of interest.

## Publisher’s Note

All claims expressed in this article are solely those of the authors and do not necessarily represent those of their affiliated organizations, or those of the publisher, the editors and the reviewers. Any product that may be evaluated in this article, or claim that may be made by its manufacturer, is not guaranteed or endorsed by the publisher.
