# Supplementary material for: High Serpin Family A Member 10 Expression Confers Platinum Sensitivity and Is Associated With Survival Benefit in High-Grade Serous Ovarian Cancer: Based on Quantitative Proteomic Analysis
Source: Front Oncol. 2021 Nov 23;11:761960. doi: 10.3389/fonc.2021.761960 (PMC8649623; doi:10.3389/fonc.2021.761960)
Supplement: Supplementary file 1 [file Presentation_1.pptx]

## Slide 1
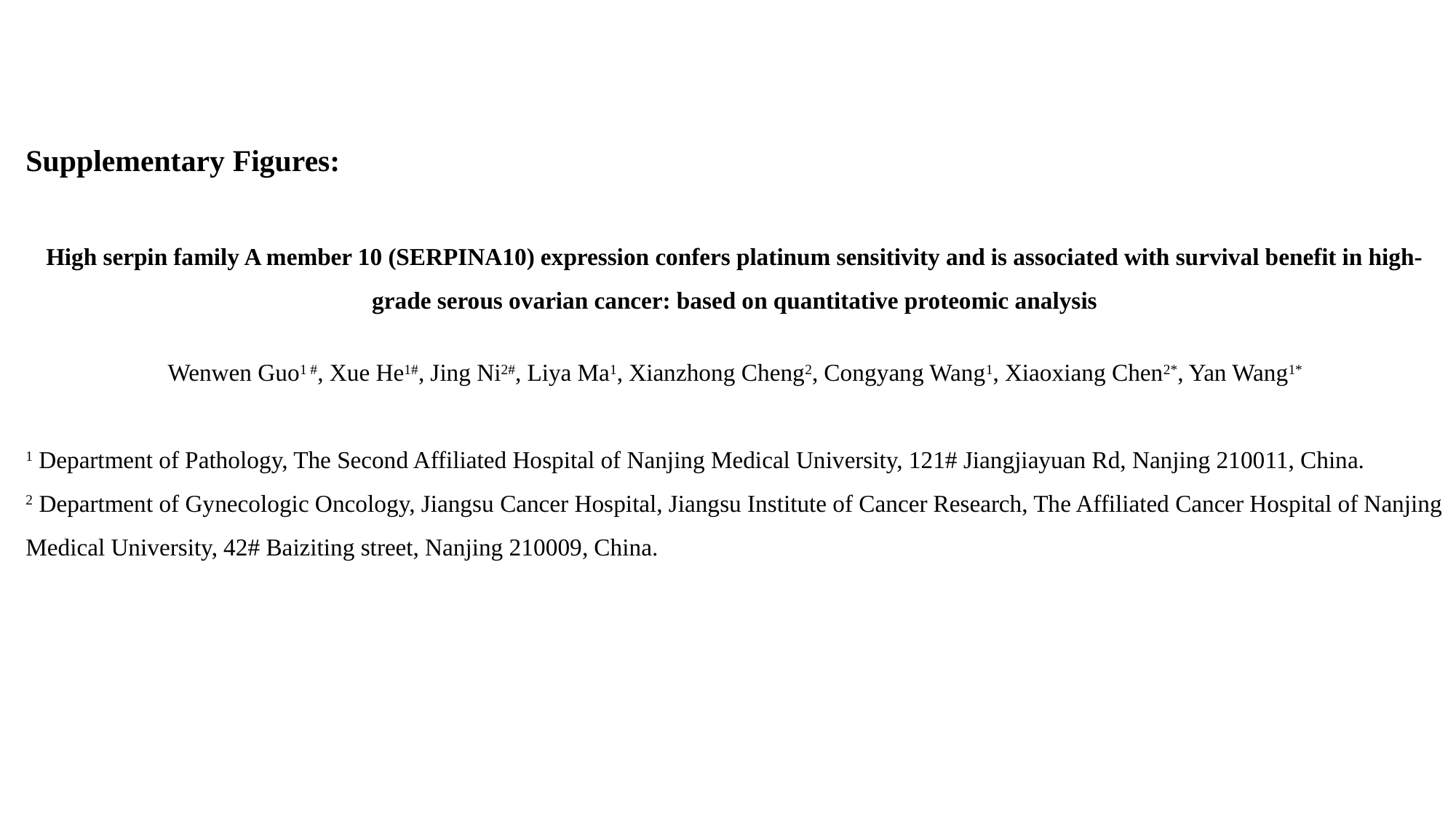

Supplementary Figures:
High serpin family A member 10 (SERPINA10) expression confers platinum sensitivity and is associated with survival benefit in high-grade serous ovarian cancer: based on quantitative proteomic analysis
Wenwen Guo1 #, Xue He1#, Jing Ni2#, Liya Ma1, Xianzhong Cheng2, Congyang Wang1, Xiaoxiang Chen2*, Yan Wang1*
1 Department of Pathology, The Second Affiliated Hospital of Nanjing Medical University, 121# Jiangjiayuan Rd, Nanjing 210011, China.
2 Department of Gynecologic Oncology, Jiangsu Cancer Hospital, Jiangsu Institute of Cancer Research, The Affiliated Cancer Hospital of Nanjing Medical University, 42# Baiziting street, Nanjing 210009, China.

## Slide 2
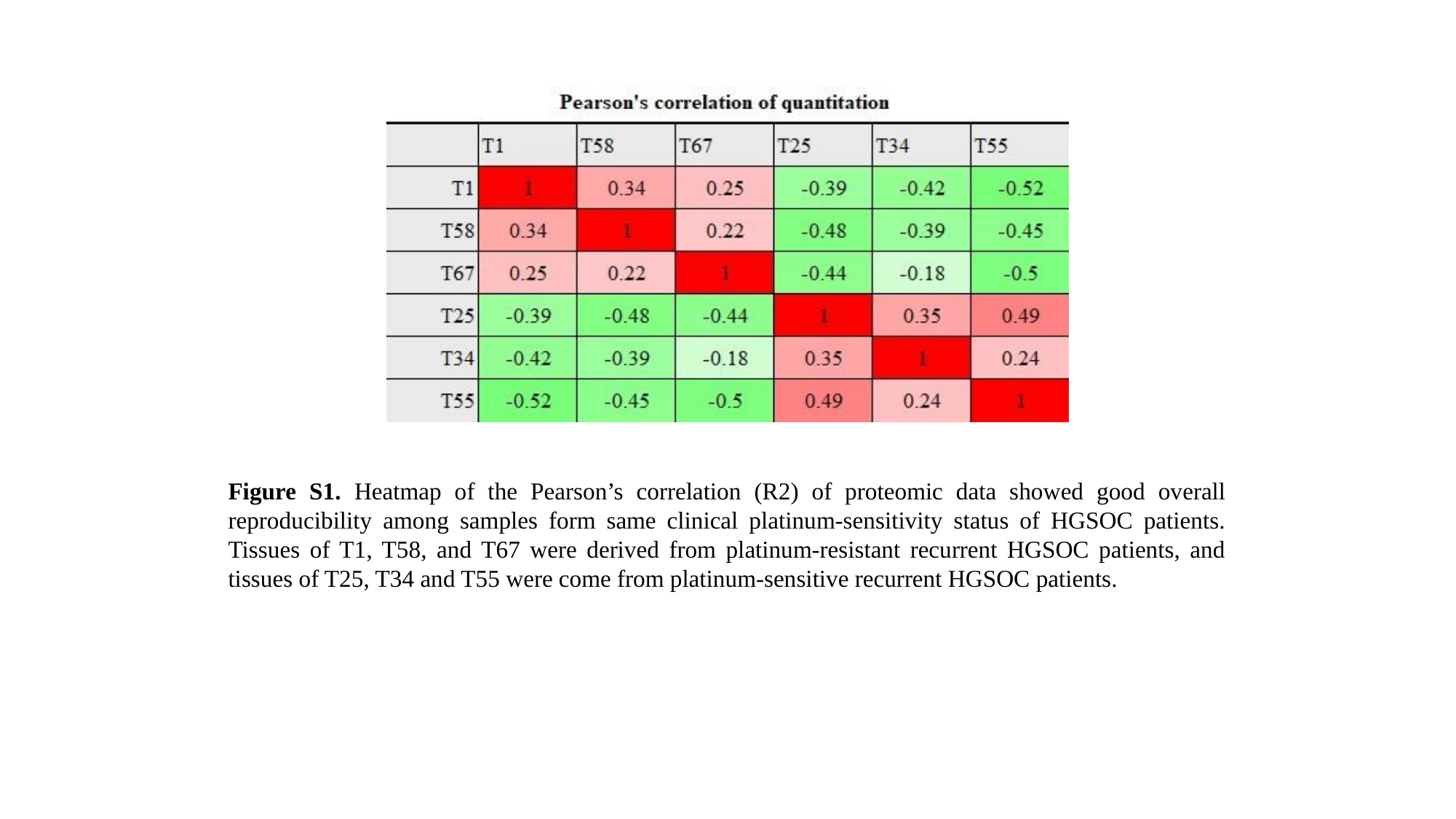

Figure S1. Heatmap of the Pearson’s correlation (R2) of proteomic data showed good overall reproducibility among samples form same clinical platinum-sensitivity status of HGSOC patients. Tissues of T1, T58, and T67 were derived from platinum-resistant recurrent HGSOC patients, and tissues of T25, T34 and T55 were come from platinum-sensitive recurrent HGSOC patients.

## Slide 3
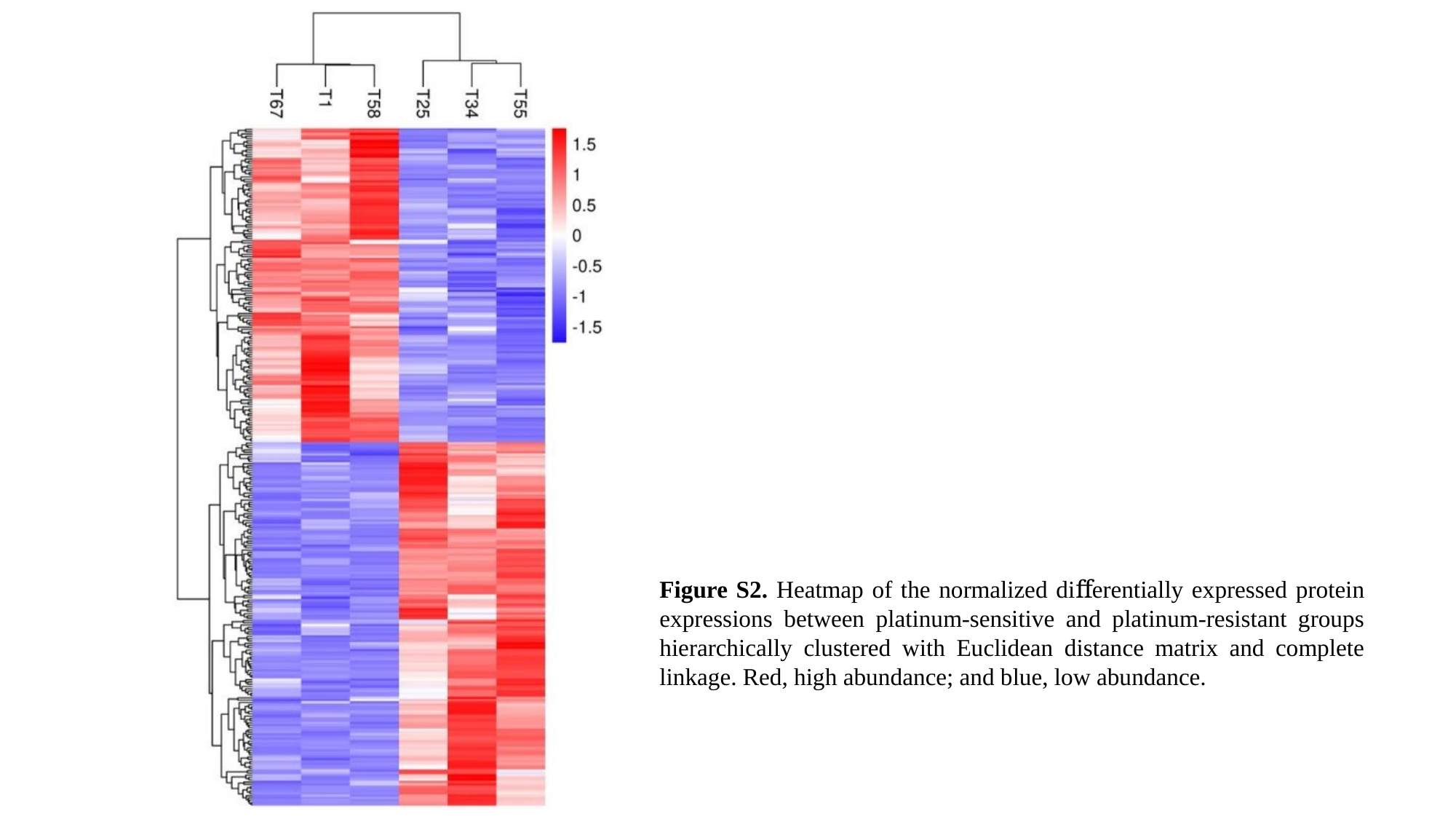

Figure S2. Heatmap of the normalized diﬀerentially expressed protein expressions between platinum-sensitive and platinum-resistant groups hierarchically clustered with Euclidean distance matrix and complete linkage. Red, high abundance; and blue, low abundance.

## Slide 4
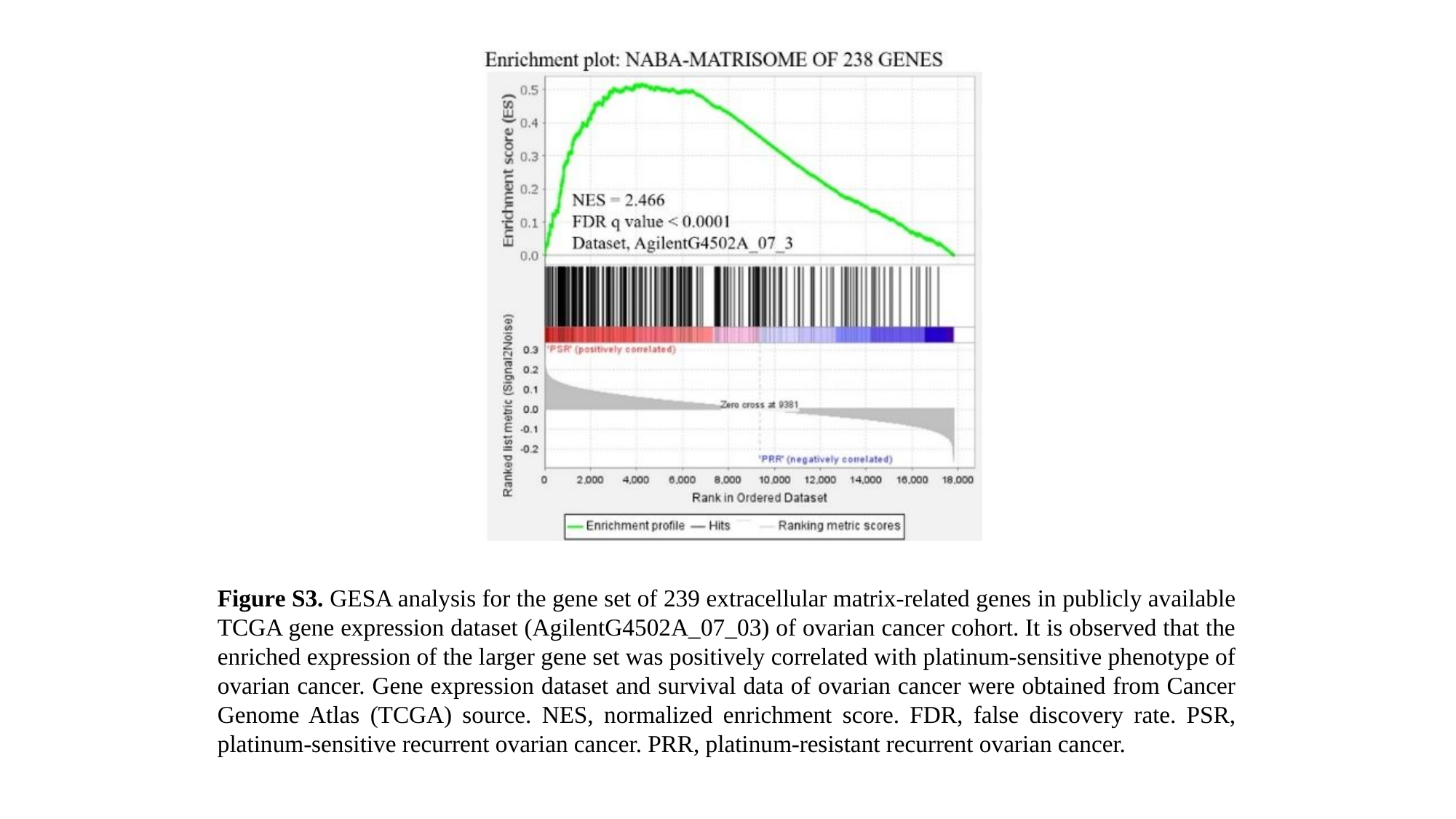

Figure S3. GESA analysis for the gene set of 239 extracellular matrix-related genes in publicly available TCGA gene expression dataset (AgilentG4502A_07_03) of ovarian cancer cohort. It is observed that the enriched expression of the larger gene set was positively correlated with platinum-sensitive phenotype of ovarian cancer. Gene expression dataset and survival data of ovarian cancer were obtained from Cancer Genome Atlas (TCGA) source. NES, normalized enrichment score. FDR, false discovery rate. PSR, platinum-sensitive recurrent ovarian cancer. PRR, platinum-resistant recurrent ovarian cancer.

## Slide 5
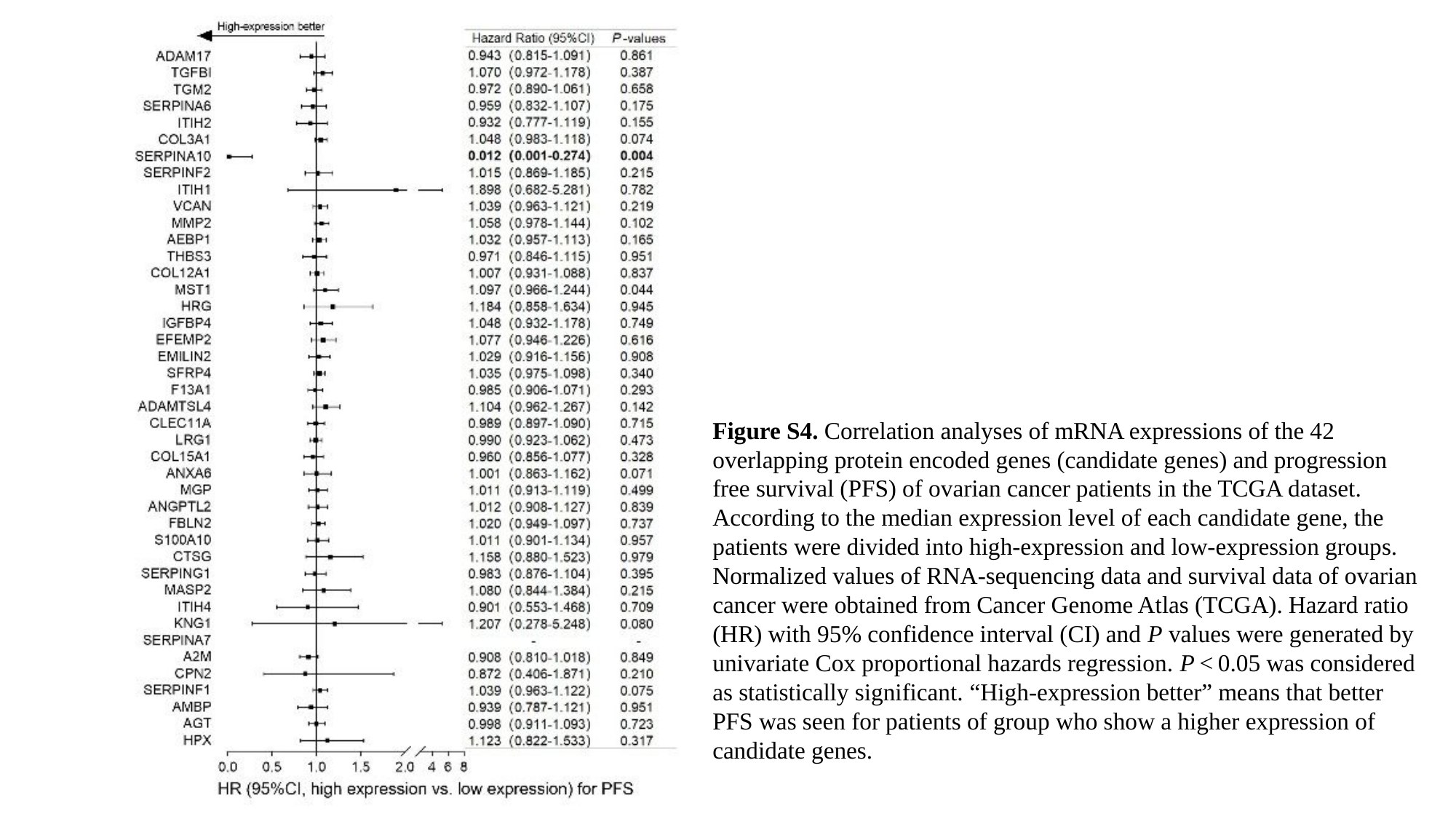

Figure S4. Correlation analyses of mRNA expressions of the 42 overlapping protein encoded genes (candidate genes) and progression free survival (PFS) of ovarian cancer patients in the TCGA dataset. According to the median expression level of each candidate gene, the patients were divided into high-expression and low-expression groups. Normalized values of RNA-sequencing data and survival data of ovarian cancer were obtained from Cancer Genome Atlas (TCGA). Hazard ratio (HR) with 95% confidence interval (CI) and P values were generated by univariate Cox proportional hazards regression. P < 0.05 was considered as statistically significant. “High-expression better” means that better PFS was seen for patients of group who show a higher expression of candidate genes.

## Slide 6
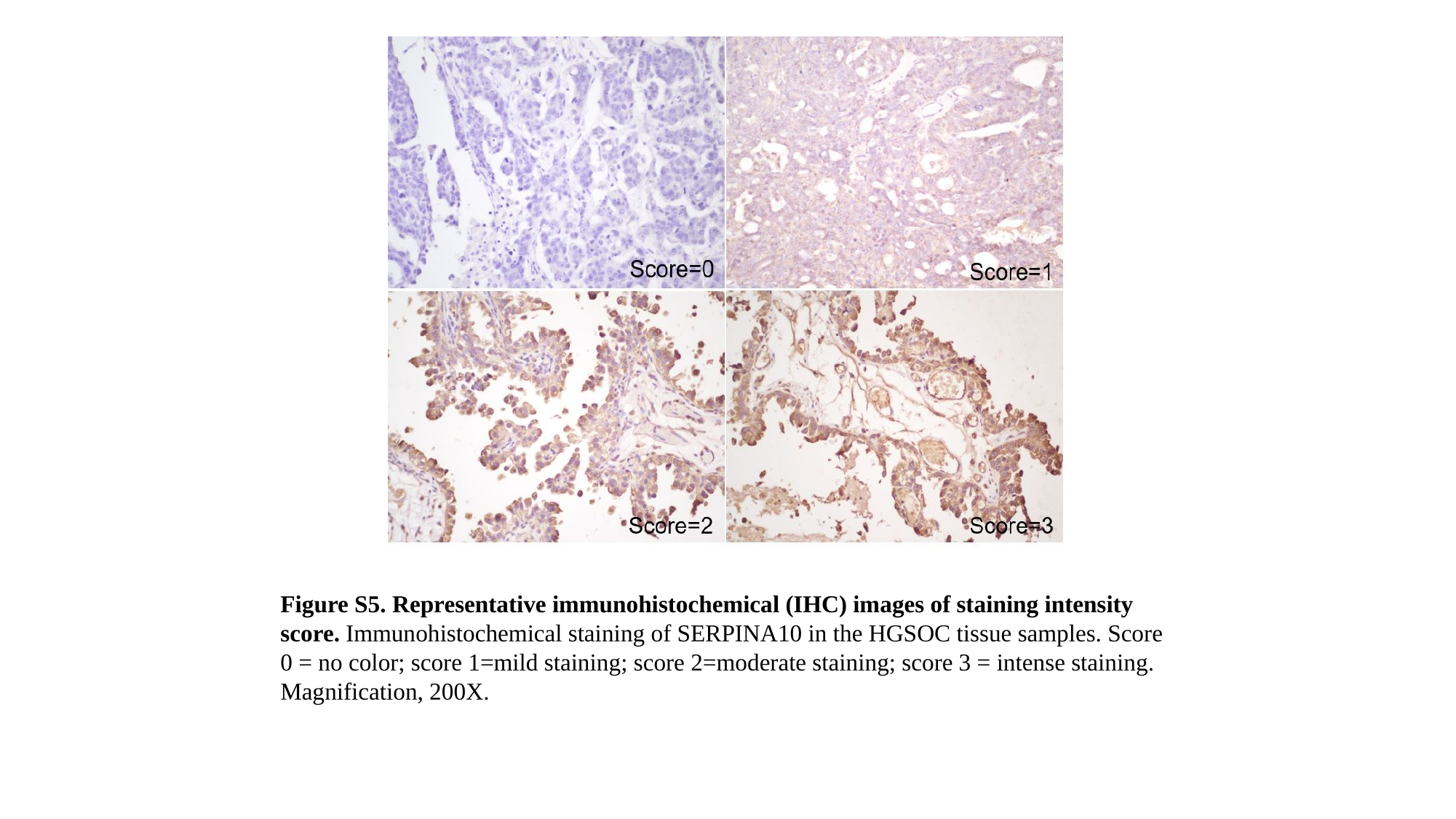

Figure S5. Representative immunohistochemical (IHC) images of staining intensity score. Immunohistochemical staining of SERPINA10 in the HGSOC tissue samples. Score 0 = no color; score 1=mild staining; score 2=moderate staining; score 3 = intense staining. Magnification, 200X.

## Slide 7
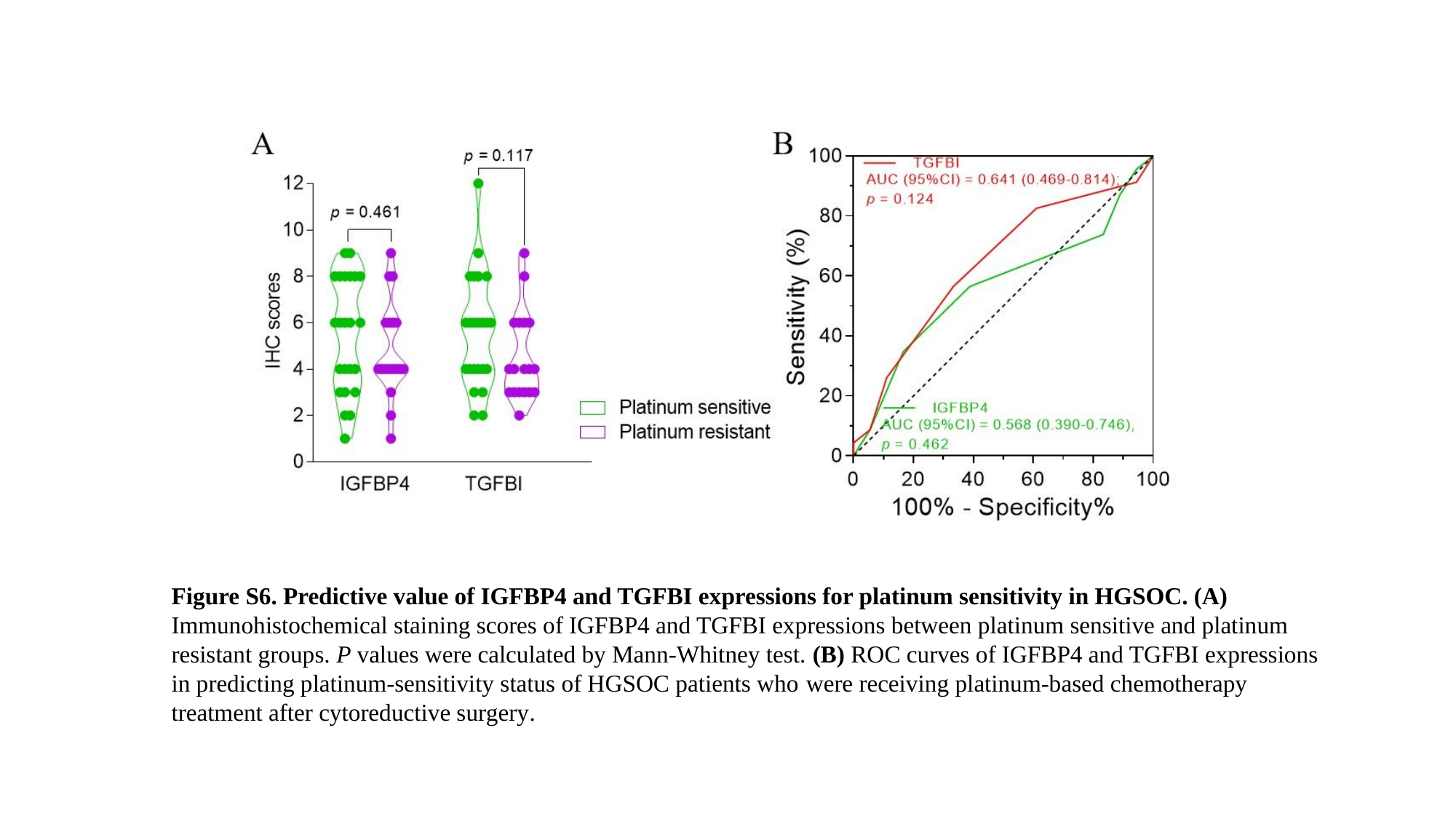

Figure S6. Predictive value of IGFBP4 and TGFBI expressions for platinum sensitivity in HGSOC. (A) Immunohistochemical staining scores of IGFBP4 and TGFBI expressions between platinum sensitive and platinum resistant groups. P values were calculated by Mann-Whitney test. (B) ROC curves of IGFBP4 and TGFBI expressions in predicting platinum-sensitivity status of HGSOC patients who were receiving platinum-based chemotherapy treatment after cytoreductive surgery.
