# Supplementary material for: High Serpin Family A Member 10 Expression Confers Platinum Sensitivity and Is Associated With Survival Benefit in High-Grade Serous Ovarian Cancer: Based on Quantitative Proteomic Analysis
Source: Front Oncol. 2021 Nov 23;11:761960. doi: 10.3389/fonc.2021.761960 (PMC8649623; doi:10.3389/fonc.2021.761960)
Supplement: Supplementary file 2 [file Table_1.docx]

Table S1. Baseline characteristics of study subjects

| Study subjects | Case ID# | Age (years) | FIGO Stage | First-line treatment | PFI (months) |
| --- | --- | --- | --- | --- | --- |
| Cohort 1 | T1 | 44 | IIIC | Paclitaxel-carboplatin | 5 |
|  | T58 | 68 | IIIC | Paclitaxel-carboplatin | 5 |
|  | T67 | 49 | IV | Paclitaxel-carboplatin | 4 |
|  | T55 | 51 | IIIC | Paclitaxel-carboplatin | 7 |
|  | T25 | 55 | IIIC | Paclitaxel-carboplatin | 29 |
|  | T34 | 66 | IIIC | Paclitaxel-carboplatin | 12 |
| Cohort 2 | S1 | 43 | III | Paclitaxel-carboplatin | 7 |
|  | S2 | 53 | IIIC | Paclitaxel-carboplatin | 7 |
|  | S3 | 51 | IV | Paclitaxel-carboplatin | 7 |
|  | S4 | 62 | IIIC | Paclitaxel-carboplatin | 8 |
|  | S5 | 68 | IIIC | Paclitaxel-carboplatin | 8 |
|  | S6 | 49 | IIIB | Paclitaxel-carboplatin | 8 |
|  | S7 | 63 | IIIC | Paclitaxel-carboplatin | 8 |
|  | S8 | 54 | IV | Paclitaxel-carboplatin | 9 |
|  | S9 | 61 | IIC | Paclitaxel-carboplatin | 9 |
|  | S10 | 59 | IIIC | Paclitaxel-carboplatin | 9 |
|  | S11 | 57 | IIIB | Paclitaxel-carboplatin | 9 |
|  | S12 | 67 | IIIC | Paclitaxel-carboplatin | 10 |
|  | S13 | 49 | IIIC | Paclitaxel-carboplatin | 11 |
|  | S14 | 69 | IIIC | Paclitaxel-carboplatin | 11 |
|  | S15 | 58 | IV | Paclitaxel-carboplatin | 12 |
|  | S16 | 68 | III | Paclitaxel-carboplatin | 12 |
|  | S17 | 54 | IIIC | Paclitaxel-carboplatin | 12 |
|  | S18 | 50 | IIIC | Paclitaxel-carboplatin | 14 |
|  | S19 | 51 | IIIC | Paclitaxel-carboplatin | 15 |
|  | S20 | 58 | IIIC | Paclitaxel-carboplatin | 16 |
|  | S21 | 55 | IIIC | Paclitaxel-carboplatin | 21 |
|  | S22 | 57 | IIIC | Paclitaxel-carboplatin | 23 |
|  | S23 | 77 | III | Paclitaxel-carboplatin | 31 |
|  | R1 | 63 | IIIC | Paclitaxel-carboplatin | 1 |
|  | R2 | 54 | IIIC | Paclitaxel-carboplatin | 1 |
|  | R3 | 69 | IIIC | Paclitaxel-carboplatin | 1 |
|  | R4 | 42 | IV | Paclitaxel-carboplatin | 1 |
|  | R5 | 58 | III | Paclitaxel-carboplatin | 2 |
|  | R6 | 51 | IIIC | Paclitaxel-carboplatin | 3 |
|  | R7 | 45 | IIIC | Paclitaxel-carboplatin | 3 |
|  | R8 | 62 | IIIC | Paclitaxel-carboplatin | 3 |
|  | R9 | 56 | IV | Paclitaxel-carboplatin | 3 |
|  | R10 | 53 | IIIC | Paclitaxel-carboplatin | 3 |
|  | R11 | 50 | III | Paclitaxel-carboplatin | 4 |
|  | R12 | 55 | IIIC | Paclitaxel-carboplatin | 4 |
|  | R13 | 51 | IIIC | Paclitaxel-carboplatin | 4 |
|  | R14 | 63 | IIIC | Paclitaxel-carboplatin | 4 |
|  | R15 | 69 | IIIC | Paclitaxel-carboplatin | 5 |
|  | R16 | 45 | IIIC | Paclitaxel-carboplatin | 5 |
|  | R17 | 51 | IV | Paclitaxel-carboplatin | 5 |
|  | R18 | 68 | IIIC | Paclitaxel-carboplatin | 5 |
